# An evaluation of the ability of *Dichelobacter nodosus *to survive in soil

**DOI:** 10.1186/1751-0147-55-4

**Published:** 2013-01-23

**Authors:** Sara Ellinor Cederlöf, Tomas Hansen, Ilka Christine Klaas, Øystein Angen

**Affiliations:** 1 Department of Large Animal Science, Faculty of Health and Medical Sciences, University of Copenhagen, Grønnegårdsvej 3, DK-1870, Frederiksberg C, Denmark; 2 National Veterinary Institute, Technical University of Denmark, Bülowsvej 27, DK-1870, Frederiksberg C, Denmark

**Keywords:** *Dichelobacter nodosus*, Soil, Real-time PCR, Survival, Bacteriological culture

## Abstract

**Background:**

*Dichelobacter nodosus* is the causative agent of footrot in sheep. The survival of the bacterium in soil is of importance for the epidemiology of the disease. The investigation evaluates the survival of *D. nodosus* in soil with and without added hoof powder stored under different temperatures.

**Results:**

An experimental setup was used with bacteriological culture and real-time polymerase chain reaction (PCR), and the results indicate that the bacteria can survive in soil for longer time than previously expected. The survival time was found to be dependent on temperature and the addition of hoof powder to the soil, with the longest survival time estimated to be 24 days in soil samples with hoof powder stored at 5°C.

**Conclusion:**

Our findings indicate that the survival time of *D. nodosus* and its ability to infect susceptible sheep on pasture under different climatic conditions should be studied further.

## Background

*Dichelobacter nodosus*, the causative agent of footrot in sheep, is a Gram-negative and obligate anaerobic bacterium [1]. The survival time for *D. nodosus* in the environment is reported to be in range of 4–14 days [1-4]. It has further been postulated that leaving hoof trimmings can increase the survival time up to six weeks [5]. The first clinical trials on survival and transmission under field conditions were made in the 1940-ies [6]. Since then, few studies have investigated the ability of *D. nodosus* to survive on the pasture. In an experimental setup viable *D. nodosus* could be cultured after 14 days [7]. Control and eradication programs are often based on the expectation that *D. nodosus* will not survive for more than a few days outside the feet of ruminants [6,8]. In Australia, a non-transmission period during the dry and hot summer period facilitates eradication [8] whereas in England, non-successful attempts to eradicate footrot have been performed during the winter, relying on the cold weather to reduce transmission and survival of the pathogen [9]. Basing control and eradication programs for one climatic region on research made in other regions can therefore be problematic. The objective of the present study was to evaluate the ability of *D. nodosus* to survive in soil in an experimental setup imitating Danish weather conditions, using culture and real-time polymerase chain reaction (PCR) to recover the pathogen.

## Materials and methods

### Soil and hoof powder

Soil from a household garden, where no sheep or other ungulates had been present, was collected into sterile plastic containers and stored at 5°C until use. The hoof powder was prepared using hooves from healthy slaughter animals, originating from a footrot free herd. Both the hoof powder and the soil were tested free from *D. nodosus* by real-time PCR before the study started. A total of 100 Eppendorf tubes were prepared with either 65 mg of soil (64.02 ± 2.76 SD) or 65 mg of soil added 10% hoof powder (63.78 ± 2.61SD). Twenty μl of sterile 0.9% saline and 15 μl bacterial suspension was added to all samples. The bacterial suspension (>10^8^ bacteria/ml) was prepared using six 4% trypticase arginine serine hoof powder (TASH) plates incubated for four days with a Danish field strain of *D. nodosus*. The soil samples were incubated aerobically at either 5°C or 15°C for 28 days creating four incubation groups of samples (soil 5°C, soil + hoof powder 5°C, soil 15°C and soil + hoof powder 15°C). The viability of *D. nodosus* was evaluated eight times over a period of 28 days; at day 4, 7, 11, 14, 17, 21, 24, and 28. On the first four days, samples were analyzed in duplicate whereas triplicates were used the last four sampling days. On all test days, the samples were chosen randomly among the incubated samples.

### Cultivation and DNA extraction

On each of the test days, the viability of *D. nodosus* was assessed through cultivation and real-time PCR. The soil samples were cultured on 4% TASH plates and incubated anaerobically (80% N_2_, 10% CO_2_ and 10% H_2_) at 37°C for four days. At day four, the colonies present were visually evaluated and the presence or absence of suspected colonies recorded. After the visual evaluation, the plates were washed with 2 ml of sterile water. The suspension was boiled for 10 min. and immediately after boiling the samples were placed on ice and centrifuged at 15.000 × *g* for 5 min. at 5°C. A 1:10 dilution of each sample was prepared. The samples were stored at −20°C until real-time PCR analysis could be performed. The remaining soil in the sample was used for DNA extraction with SoilMaster™ DNA extraction kit (Epicentre® Biotechnologies, Madison, WI, USA) according to the protocol of the manufacturer [10]. The following modifications were made to the protocol: the optional step 3 and step 15 in the cell lysis procedure was excluded. In step 10, 125 μl of supernatant was used, and in step 16, 100 μl of TE buffer was used to re-suspend the DNA. The prepared templates were stored at – 20°C until real-time PCR analysis could be performed.

### Real-time PCR

All templates were thawed at room temperature before analysis. *D. nodosus*-specific PCR primers and probes targeting the 16S rRNA gene were used. The test was performed as earlier described [11] on undiluted and 1:10 diluted samples. In all runs, two positive and two negative controls were included. As positive control the type strain of *D. nodosus* (CCUG 27824) was used in a 1:100 dilution, while sterile 0.9% saline was used as NTC.

### Analysis of results

Fluorescence signals were analyzed using a manually set threshold at 0.01 in the software Rotor-Gene Q Series Software (v. 2.0.2.). Outlier removal was performed using 10%, and slope correction was used. For all calculations, results from the 1:10 diluted samples were used in order to reduce bias from inhibition. A cycle threshold (Ct) value cut-off at 37 was used to define a positive sample A Ct-value cut-off of 25 was set to separate viable from non-viable *D. nodosus* present on the agar plates. This cut-off was based on testing the same amount of soil as used for incubation of agar plates dissolved in 200 μl sterile water. Soil was tested this way for all soil types on day 21 and day 32 respectively. The test revealed that this amount of soil only resulted in Ct-values above 25 (for most samples >30) in the 1:10 dilution.

### Statistics

All statistical calculations were done using SAS statistical software version 9.2 (SAS Institute Inc., Cary, NC, 2004) on a 5% significance level. The proportion of samples with Ct-values > 25 or ≤ 25 within and between each of the four incubation groups were compared with Proc Freq and Fishers exact test, in order to determine differences between test days. For each of the four groups, the survival time was defined as the last test day, where at least one of the samples obtained a Ct-value below the cut-off of 25. The Ct-values from the real-time PCR analysis of DNA extracted from soil via the SoilMaster™ DNA extraction kit were compared between test days for each soil type, using a Kruskal-Wallis test to test for significant variation in the amount of DNA extracted.

## Results

At day 4, the four incubation groups showed similar median Ct-values based on plate of culture (Table 1). In general a lower temperature was associated with a lower Ct-value and groups containing hoof powder had lower Ct-values compared to pure soil samples incubated at the same temperature (Table 1). Using a Ct-value cut-off of 25, a significant difference within and between the four groups at different test days could be found (*P* = 0.005). Viable bacteria, defined as Ct-values below 25, were found to be present until day 14 for soil at 5°C and until day 24 for soil + hoof powder at 5°C. A marked reduction in survival time could be seen for samples stored at 15°C; viable bacteria could only be retrieved until day 4 from pure soil and until day 7 for soil + hoof powder. Furthermore, an association could be seen between recorded suspected colonies on agar plates and Ct-values of ≤ 25 (*P* < 0.0001) (Table 1). Attempts were made to isolate the bacteria from day 17 and until the end of the experiment. No pure cultures could however be obtained. DNA could be retrieved from all groups throughout the experiment with SoilMaster™ DNA extraction kit, and the variation of the Ct-values within the groups between test days was small and a significant variation was only observed between soil with and without hoof powder stored at 5°C (*P*=0.04). The pure soil stored at 15°C had the highest Ct-values.

**Table 1:** **Results based on plate wash of cultures from the four groups of samples, presented on the different test days with Ct-values and observation of *Dichelobacter nodosus*-suspect colonies**

	**Soil 5°C**		**Soil and hoof powder 5°C**		**Soil 15°C**		**Soil and hoof powder 15°C**	
**Day**	**Ct-value**^**a**^	**Colonies**^**b**^	**Ct-value**	**Colonies**	**Ct-value**	**Colonies**	**Ct-value**	**Colonies**
4	17.19	Y	17.53	Y	18.83	N	18.47	Y
	17.28	Y	18.15	Y	17.12	Y	15.52	Y
7	18.08	Y	18.52	Y	34.02	Y	22.68	N
	18.64	N	16.91	Y	34.08	N	22.02	N
11	21.60	N	19.42	Y	33.34	N	31.15	N
	25.15	N	18.24	Y	33.97	N	32.13	N
14	24.04	Y	16.51	Y	35.87	N	31.77	N
	22.50	N	18.95	Y	30.87	N	45	N
17	29.95	N	19.98	Y	45	N	29.88	N
	25.10	Y	17.31	Y	45	N	30.64	N
	25.45	N	18.16	N	45	N	29.26	N
21	45	Y	24.92	Y	45	N	32.39	N
	45	Y	34.51	Y	45	N	31.65	N
	26.41	N	45	Y	45	N	33.07	N
24	36.57	Y	32.25	N	45	N	36.11	N
	34.23	N	19.27	N	45	N	31.87	N
	35.19	N	25.45	Y	45	N	31.56	N
28	45	N	36.07	N	45	N	31.63	N
	34.83	N	31.26	N	45	N	30.71	N
	45	N	32.57	N	45	N	33.00	N

^a^: All Ct-values presented are for the 1:10 diluted samples. On day 4–14 samples were analyzed in duplicates whereas triplicates were used from day 17 to 28.

^b^: Observed *D. nodosus*-suspect colonies on TASH agar plates (Yes/No).

## Discussion

Non-sterile soil was chosen to mimic the survival conditions for *D. nodosus* in the field. Hoof powder was added for several reasons. The standard cultivation medium for *D. nodosus* contains hoof-powder as this has been shown to increase the growth of the bacterium. In addition, when *D. nodosus* is released from the hooves this can be expected to happen together with sloughed-off pieces of horn. Another possible situation could be that residues from hoof-trimming were left on the field

We were not able to obtain pure cultures of *D. nodosus* from the soil samples due to the heavy growth of soil bacteria on the TASH agar plates. However, an association was found between the observation of colonies resembling *D. nodosus* and low Ct values from plate wash, indicating the presence of growing of *D. nodosus.* qPCR will detect both living and dead bacteria as well as free DNA in a sample but can nevertheless under certain circumstances be used for assessing the viability of bacteria. Enrichment of bacteria in a growth medium prior to qPCR has been used in several publications, e.g. for measuring viability of *Escherichia coli* O157:H7 in drinking water [12]. When the measured Ct values are lower than what can be expected from the residual DNA used for inoculating the media it can be concluded that this must be dependent on the growth of the bacterium in the enrichment medium. A Ct cut-off value of 25 was chosen, as our experiments showed that the amount of *D. nodosus* DNA in the soil used for inoculating the TASH agar plates gave Ct values well above this value. Uyttendale *et al*. [13] found that false positive qPCR results (results due to non viable bacteria) were only observed when an amount of DNA corresponding to more than 10^8^ colony forming units (CFU)/ml *E. coli* was inoculated into the enrichment medium. For comparison, the starting concentration of *D. nodosus* in the soil samples was approximately 10^7^ CFU/ml. In soil samples, degradation of DNA can proceed rapidly due to numerous nucleases [14]. Lebuhn *et al.*[15] found that qPCR was an acceptable method to estimate bacterial viability in samples having a substantial metabolic turnover (as soil samples) and that DNA from decaying organisms was readily recycled in metabolically active environments. Romanowsi *et al*. [16] investigated the transforming activity of plasmid DNA in soil and found that it dropped below the detection level after 10 days. The soil samples used in the present investigation showed a considerable microbiological activity as seen by the growth on the TASH agar plates. Furthermore, rapid decrease of the PCR signal in the soil sample kept at 15°C obviously reflects the decay of both *D. nodosus* cells and DNA. Nevertheless, amplifiable *D. nodosus*-DNA was found to be present in all samples after DNA extraction although in smaller amounts in pure soil samples stored at 15°C. Based on these considerations, it seems safe to conclude that Ct values below 25 reflect that viable *D. nodosus* cells were present at the day of cultivation.

## Conclusions

Ct-values below 25 found at day 14 and 24 indicate that *D. nodosus* can survive in soil for longer time than previously expected. Decreased temperature and the addition of hoof powder to soil increased the survival time. Real-time PCR on a similar amount of soil as used for inoculation of agar plates gave all Ct-values above 25 which consequently represent a conservative threshold for separating viable from non-viable bacteria. Ct-values below 25 were detected up to day 24 in the samples containing hoof powder stored at 5°C. Our findings indicate that the survival time of *D. nodosus* and its ability to infect susceptible sheep on pasture under different climatic conditions should be studied further.

## Competing interests

The authors declare that they have no competing interests.

## Authors’ contributions

SEC designed the study, performed the experimental work and the statistical analyses, and drafted the first version of the manuscript. TH designed the study and performed the experimental work and statistical analyses. ICK supervised in the design of the study and the statistical analysis. ØA supervised in the design of the study and the experimental work. All the authors read and approved the final manuscript.
